# Physical therapy rehabilitation after hospital discharge in patients affected by COVID-19: a systematic review

**DOI:** 10.1186/s12879-023-08313-w

**Published:** 2023-08-16

**Authors:** Perez A. M. C., Silva M. B. C, Macêdo L. P. G., Chaves Filho A. C., Dutra R. A. F, Rodrigues M. A. B.

**Affiliations:** 1 Department of Biological Sciences, Federal University of Pernambuco, Recife, Brazil; 2 Department of Electronics and Systems, Federal University of Pernambuco, Recife, Brazil; 3 Department of Biomedical Engineering, Federal University of Pernambuco, Recife, Brazil

**Keywords:** Rehabilitation, Physical Therapy Modalities, Covid-19, Post-acute Syndromes COVID-19, Physical Fitness

## Abstract

In 2019, the Coronavirus (SARS-CoV-2) virus was identified, also defined as novel coronavirus or severe acute respiratory syndrome coronavirus 2. The Corona VIrus Disease (COVID-19) pandemic generated several changes in society in 2020 as a result of its rapid spread in humans. The main symptoms are high fever (≥ 37.8 °C), dry cough, dyspnea, upper respiratory symptoms, myalgia, fatigue and diarrhea in the most uncommon cases. Considering the emergency situation caused by the COVID-19 pandemic and the effects attributed to it, rehabilitation professionals have a fundamental role in the functional recovery of patients, independence and improvement of quality of life. This is a systematic review of the literature, with the aim of discussing the main findings on physical therapy management in functional changes in post-COVID-19 patients. Pubmed, Scielo, Sciencedirect, BVS and PEDro databases were used. The terms MESH/DECs used for the searches were: Rehabilitation, Physical Therapy Modalities, Covid-19, Post-acute Syndromes COVID-19 and Physical Fitness, the keywords were also used: rehabilitation, physiotherapy, Covid-19, post- acute syndrome COVID-19 and functional capacity. To cross the terms, Boolean operators (AND and OR) were used. Randomized trials, recommendations, quasi-randomized or prospective controlled trials, reports, guidelines, and field updates were included. As for the selected population, studies were included in individuals of both sexes, with no age restriction, that evaluated physiotherapeutic interventions in patients who had COVID-19. Literature reviews, case studies, conferences, abstracts of articles published in conference proceedings and letters to the editor were excluded from the research. To measure methodological quality, the PEDro scale was used.Searches for articles were performed restricting the period of publication between the years 2019 to 2022. The electronic search strategy identified a total of 364 records from the selected databases. After screening for duplicates, 14 articles were excluded, followed by screening by titles and abstracts, another 298 articles were excluded, of these 47 potentially relevant records were submitted to full text review and of these 5 randomized clinical trials were included in this review. In view of the findings of this study, it can be concluded that physical therapy rehabilitation should continue after hospital discharge, with the aim of improving physical performance and activities of daily living (ADL) in post-COVID-19 patients.

**Supplementary Information:**

The online version contains supplementary material available at 10.1186/s12879-023-08313-w.

## Introduction

COVID-19 is an infectious disease caused by severe acute respiratory syndrome coronavirus 2 (SARS-CoV-2) [1]. The Pandemic greatly impacted the world scenario, aggravating morbidity and mortality rates [2]. The disease was responsible for many severe cases, mainly in the respiratory system, accompanied by fever, muscle pain and fatigue, even though it is a systemic disease [3, 4]. The virus has high transmissibility between humans, which further aggravated the disease control condition [5].

In addition to respiratory and musculoskeletal changes, the absence of smell and taste can occur as an indication of infections in the early stages of the disease [5]. Studies report that COVID-19 has characteristics similar to common flu conditions, but in some cases, it can lead to serious manifestations and even lead to death, especially in the elderly population and in individuals with chronic comorbidities, being considered a pandemic and emergency of disease public health of international interest [6].

Considering the emergency situation caused by the pandemic, and the deleterious effects not yet fully known, some measures must be taken in view of the possible effects attributed to the disease, such as: motor, respiratory and hemodynamic changes that may also be related to the length of hospital stay and of bed restriction. It is estimated that 5% to 10% of patients are indicated for a post-COVID-19 rehabilitation program [7]. In this way, physical therapy rehabilitation has a fundamental role in the recovery and restoration of the motor functions of people affected by the disease [8].

Some factors can compromise the patient's rehabilitation protocol, such as the availability of rehabilitation services, the difficulty in moving and accessing rehabilitation units, and the patients' financial situation, among others [9]. Rehabilitation professionals play an important role throughout the disease evolution process, from installation, participating in intensive care until discharge, favoring the return of these individuals to their homes and providing continuity of care at that moment [3].

The physiotherapy protocol should be continued after hospital discharge, as there are reports of low physical functioning and impaired performance in activities of daily living in post-COVID-19 patients [10]. The physiotherapy protocol should be individual, focusing on the deficits presented by the individual, aiming to minimize the risks and problems bringing positive impacts on improving functionality, providing independence to the individual, favoring the improvement of the quality of life [8, 11, 12].

This article had as main objectives, to discuss the effects of the main findings on the physical therapy management in the functional alterations of post-COVID-19 patients, to observe the benefits of Physical Therapy in the rehabilitation of patients affected by COVID-19 after hospital discharge, to verify the existence of physiotherapeutic protocols for post-COVID-19 rehabilitation and to evaluate the main functional sequelae in post-covid-19.

## Methods

### Record

The protocol for this review was registered in the International Prospective Register for Systematic Reviews (PROSPERO), under registration number: CRD42023401750.

### Study design

This systematic review aimed to discuss the main findings on physiotherapeutic management of the main functional changes in post-COVID-19 patients after hospitalization discharge. The study was edited following the PRISMA checklist.

### Data extraction

Information about the chosen works was summarized in a standard form, organized in order to extract the data considered relevant from each of the studies. The objective was to structure the data and ensure consistency in the extraction process. The information extracted from each study was: general information (author, year of publication, objective, sample and results). In the data extracted for the systematic review, the results of the articles were presented according to the original articles. In the topic results, Table 1. there is a summary of the studies selected for the systematic review.

**Table 1:** Number of articles extracted from each database

Bases	Number	excluded by duplicity	excluded by title	excluded by summary	full text excluded	accepted
PubMed	174	2	128	25	17	2
Scielo	6	1	1	1	3	0
Science Direct	132	5	72	32	22	1
BVS	31	3	14	9	4	1
PEDro	21	3	14	2	1	1
Total	364	14	229	69	47	5

### Research strategy and selection of studies

A systematic literature search was carried out from January 2021 to June 2022 in Pubmed, Scielo, Sciencedirect, BVS and PEDro databases. Searches for articles were performed restricting the period of publication between the years 2019 to 2022. The keywords used were:Rehabilitation, Physical Therapy Modalities, Covid-19, Post-acute Syndromes COVID-19**,** Physical Fitness. To cross the terms, the Boolean operators (AND and OR) were used. For data extraction, the above-mentioned keywords were used. Next, Table 1 shows the number of articles extracted from each database.

### Eligibility criteria

Only studies that investigated the physical therapy management of the main functional, such as: Changes in muscle strength, balance, fatigue, gait deficit, changes in activities of daily living and limitation of range of motion, changes in post-Covid-19 patients were defined as eligibility criteria. Randomized trials, recommendations, quasi-randomized or prospective controlled trials, reports, guidelines and field research were included. As for the selected population, studies involving individuals of both sexes were included, with no age restriction. Literature reviews, case studies, conferences, abstracts of articles published in conference proceedings and letters to the editor were excluded from the research.

### Study selection procedures

The selection of studies took place in three stages: search and identification of studies, removal of duplicates and reading of articles. The stage of searching the databases consisted of obtaining titles after performing the searches in the databases and applying filters, such as: publication period, publication language and type of publication, as well as the analysis of eligibility criteria. The step of removing duplicates consisted of searching for duplicate references in the totality of titles obtained, through the Mendeley reference manager, after this verification, only one title was kept and its copies were excluded. Two independent reviewers checked full texts using titles and abstracts. Studies that met our eligibility criteria were included in this review and when there were discrepancies between reviewers these were resolved by a third reviewer.

### Assessment of the methodological quality of the studies

The evaluation of the methodological quality of the selected articles was performed using the PeDro scale. The scale consists of 11 questions about the study, of which 10 are scored, each item corresponds to 1 point when it reaches its objective, with the exception of item 1, which is not scored. Thus, the total score can vary from 0 (zero) to 10 (ten) points, where 9–10 points correspond to excellent quality, 8–9 points correspond to good quality, 4–5 points correspond to reasonable quality and below 3 points are classified as studies of low methodological quality. Studies with a PEDro scale ≥ 4 points were included in the present study,indicating an acceptable quality for inclusion [13]. The PEDro scale is composed of the following criteria: 1) specification of the inclusion criteria (unscored item); 2) random allocation; 3) allocation secrecy; 4) similarity of groups in the initial or baseline phase; 5) masking of subjects; 6) masking of the therapist; 7) blinding of the evaluator; 8) measurement of at least one primary outcome in 85% of the allocated subjects; 9) intention-to-treat analysis; 10) comparison between groups of at least one primary outcome and 11) reporting of variability measures and estimation of parameters of at least one primary variable.

### Outcome measures

The primary outcome was physical therapy rehabilitation on functional changes in post-COVID-19 patients measured by comparing different physical therapy protocols, between remote and face-to-face physical therapy protocols, or physical therapy protocols compared with no protocol, and the results were measured using scales and assessment tests, such as: Mini BestTest and the Timed Up and Go, Post-COVID-19 Functional Status Scale questionnaire, Borg scale. We organized treatment effects into three categories: (1) Physiotherapeutic protocols for post-COVID-19 rehabilitation; (2) Benefits of Physiotherapy for post-COVID-19 rehabilitation; (3) main post-covid-19 functional sequelae.

### Analysis of results and data

The variables analyzed in the articles were performed from the main outcome addressed in this study and are addressed in the form of a table, described according to the evaluated categories. Possible relationships between the included studies were clarified throughout the text, explaining their characteristics. It was not possible to carry out a meta-analysis, due to the heterogeneity of the information found and the incompleteness of the data, the results were presented as descriptive data.

## Results

In this section, the results obtained in this research will be presented, after reading all the articles eligible for analysis.

The analyzed population consisted of participants of both sexes in the post-COVID-19 rehabilitation process, with functional alterations (deficit in gait, strength, balance or reduced quality of life). The study included 302 adults, aged between 18 and 75 years, it was not possible to quantify how many men and how many women participated in the studies respectively, who were diagnosed with COVID-19.

One study assessed functional capacity through the 6-min walk test Li et al. (2022) [14]. Another study PEHLIVAN et al., (2022) [15] investigated the functional performance of participants through a battery of short-term tests: 30-s sit-to-stand test, lower limb strength, baseline visual analogue scale fatigue score, and modified dyspnea score. No significant differences were found in the performance tests, however the visual analogue scale fatigue score was similar, the mMRC score was higher in the TG (*p* = 0.012).

Another study, Nambi et al. (2022) [16], evaluated muscle strength, muscle mass and quality of life. strength was measured with a portable dynamometer (Camry digital hand dynamometer, EH 101–17). Quality of life: It was subjectively measured using the Sarcopenia and Quality of Life (SarQol) questionnaire. After high and low intensity exercise. Strength improved in the handgrip strength group improved more (*P* < 0.001) in the low-intensity aerobic training group than in the high-intensity aerobic training group, but not in the amount of muscle. Quality of life showed greater improvement (*P* < 0.001) in the low-intensity group than in the high-intensity group.

Outro estudo, Foged et al. 2021 [17], realizou um protocolo intervalado de alta intensidade (HIIT), Foi utilizado três protocolos, com duração do exercício (38 min). Um aquecimento de 10 minutos foi seguido por um bloco de exercícios intervalados, com duração entre 21 e 25 minutos dependendo do protocolo específico, com o objetivo de avaliar a tolerância do exercício em pacientes pó-COVID-19. Outro estudo, Giardina et al. (2022), avaliaram o equilíbrio com uma plataforma estabilométrica, enquanto o equilíbrio dinâmico foi avaliado com o Mini BESTest e o teste Timed Up and Go.

Another study, Foged et al. 2021 [17], performed a high-intensity interval protocol (HIIT), three protocols were used, with exercise duration (38 min). A 10-min warm-up was followed by an interval exercise block, lasting between 21 and 25 min depending on the specific protocol, with the aim of assessing exercise tolerance in post-COVID-19 patients. Another study, Giardina et al. (2022) assessed balance with a stabilometric platform, while dynamic balance was assessed with the Mini BESTest and the Timed Up and Go test.

Regarding the protocols used, two articles by Giardinia et al. (2022) and Nambi et al. (2022) [16], provided a description of the intervention groups, types of therapy and form of application. The article by Giardinia et al. (2022) showed that there was a significant difference in the time taken to perform the TUG test between groups (*P* < 0.0001); in post-hoc, PwCOVID and PwAECOPD did not differ significantly (*P* = 0.274), while healthy subjects performed better than PwCOVID (*P* < 0.0001) and PwAECOPD (*P* = 0.008). As for the Mini-BESTest, and also in the TUG test, the effect size of the differences between post-COVID-19 and healthy individuals was greater than 0.8. The article by Nambi et al. (2022) [16], showed that after 6 months of intervention there was a significant improvement related to strength and quality of life in the low-intensity exercise group compared to the high-intensity group, with p value. 0.003. Three other studies, PEHLIVAN et al., (2022) [15], Foged et al. 2021 [17] and Li et al. (2022) [14] did not provide the description of the intervention groups, nor types of therapy nor how they were applied. The sample was calculated in only one article Foged et al. 2021 [17].

Due to the heterogeneity of clinical trials in terms of types of protocols, participant characteristics, interventions and comparison groups, the types of protocols used in the functional rehabilitation of post-COVID-19 individuals remain uncertain. In addition, most studies analyzed some of their outcomes based on statistical significance, but it was not possible to calculate the magnitude of the treatment effect.

### Search results

**Fig. 1:**
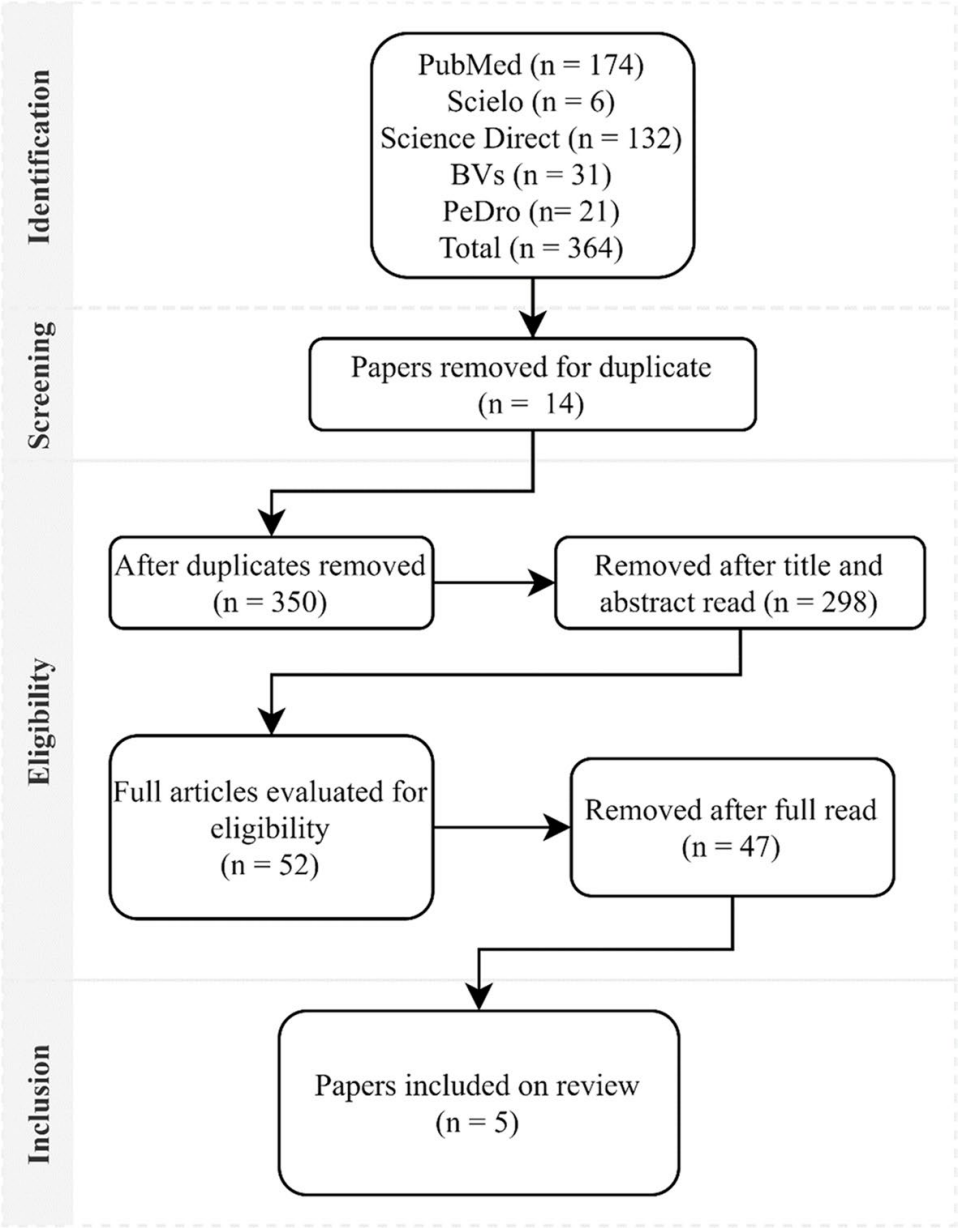
Review search strategy flowchart

The electronic search strategy identified a total of 364 records from the selected databases. After screening for duplicates, 14 articles were excluded, followed by the screening by titles and abstracts, another 298 articles were excluded, of these 47 potentially relevant records were submitted to full text review and of these, 5 articles of a scientific nature, of the type essays Randomized clinicians were included for the qualitative synthesis of this review. The detailed flowchart of the search strategy is shown in Fig. 1.

The Kappa score was used to evaluate the extracted data and verify the agreement between the evaluators regarding the inclusion and exclusion of the studies. it was observed that evaluator 1 decided to include 10 articles and evaluator 2 to include 7, and for 5 articles there was a concordant decision on inclusion. The non-inclusion results were concordant for 352 articles. For 7 articles there was disagreement for inclusion, and for 5 articles evaluator 1 decided for inclusion and evaluator 2 did not, and for 2 articles evaluator 2 decided for inclusion and evaluator 1 did not. The results of this analysis are shown in Table 2.

**Table 2:** Kappa agreement values

Studies	Feature evaluated	Cohen’s K	IC95%	Result
364	Decision for inclusion/exclusion	0,5,788,876,276,957,998	98.11320754716981%	moderate concordance

### Main findings of selected studies

The eligible clinical studies selected for this systematic literature review were characterized according to the following variables analyzed: year of publication of the study, objective, sample size, intervention and main results found in each study, as shown in Table 3.

**Table 3:** Summary of the studies selected for this systematic review on the physiotherapeutic management of the main functional changes after covid-19

Author/Year	Aim	Sample	Intervention	Outcome measures	Results
Li et al. (2022) [14]	To investigate the superiority of a telerehabilitation program for COVID-19 over no rehabilitation	Total: 120 GE: 59 GC: 61	GE: Performed 6-week unsupervised home exercises, delivered via a smartphone app called RehabApp GC: Received short educational instructions at baseline	6-min walk test (6MWT), squat in seconds; lung function assessed by spirometry; HRQoL measured with Short Form Health Survey-12 (SF 12) and mMRC-dyspnoea	This study demonstrated the superiority of TERECO over no rehabilitation for the outcomes analyzed
PEHLIVAN et al., (2022) [15]	examined the effectiveness of some online performance tests on Covid-19 cases in showing the physical performance changes of cases	Total: 21 GE: 11 GC: 10	The EG received breathing exercises, active breathing cycle techniques, range of motion and light aerobic exercises. The GC, on the other hand, received a leaflet with information about basic exercises that can be done at home and about the disease	30-s sit-to-stand test; short-term performance test; Consultation of fatigue on visual analogue scale and dyspnea scale (mMRC)	The authors reported that an online exercise program has positive effects on the physical performance of Covid 19 cases
Nambi et al. (2022) [16]	To compare the clinical and psychological effects of low-intensity and high-intensity aerobic training combined with resistance training in community-dwelling elderly men with symptoms of post-CONVID-19 sarcopenia	Total: 76 G1: 38 G2: 38	G1: Received low-intensity aerobic training for eight weeks G2: received high-intensity aerobic training for eight weeks	Muscle quantity: magnetic resonance imaging; Hand grip strength: portable dynamometer (Camry digital hand dynamometer, EH 101–17);(RM)Quality of life: Sarcopenia and Quality of Life questionnaire (SarQol); Kinesiophobia: The Tampa Scale of Kinesiophobia – 11; perceived exertion (RPE); (Borg scale 6–20) and Likert scale	Low-intensity aerobic training exercises improved clinical and psychological aspects compared to high-intensity aerobic training in older adults with post-COVID-19 sarcopenia
Foged et al. 2021 [17]	Investigate the fidelity, tolerability and safety of three different training protocols high intensity interval (HIIT) in individuals who have been hospitalized due to COVID-19	Total: 10	A randomized crossover trial was performed to compare three supervised HIIT protocols (4 × 4, 6 × 1, 10–20-30) in 10 subjects who were recently discharged after hospitalization for severe COVID-19	Muscle quantity: magnetic resonance imaging; Hand grip strength: portable dynamometer (Camry digital hand dynamometer, EH 101–17);(RM)Quality of life: Sarcopenia and Quality of Life questionnaire (SarQol); Kinesiophobia: The Tampa Scale of Kinesiophobia – 11; perceived exertion (RPE); (Borg scale 6–20) and Likert scale	The authors suggest that individuals who have been hospitalized for severe COVID-19 can safely tolerate the HIIT protocol as a rehabilitation strategy in this context
Giardinia et al. (2022)	To assess balance in patients with post-acute COVID-19 compared with patients with acute exacerbation of chronic obstructive pulmonary disease and healthy subjects	Total: 75 G1: 25 G2: 25 G3: 25	All individuals underwent specific tests to assess balance and, at the end, comparative tests were performed between the groups	Mini Balance Evaluation Systems Test (Mini-BESTest) and Timed Up and Go (TUG),	The study demonstrated that there were similar results between COVID and PwAECOPD. PwCOVID showed a balance deficit in both dynamic and static conditions

*GC* Control Group, *GE* Experimental Group, *G1* Group 1, *G2* Group 2, *G3* Group 3

### Results of the evaluation of the methodological quality of the studies using the PEDro scale

The articles were grouped according to the methodological quality assessment, according to the analysis of the PEDro scale items. According to the PEDro scale classification, one study reached 8 points, two studies reached 7 points, one reached 6 points and one reached 5 points. No studies reported conflicts of interest or funding related to commercial interests. The items that verify the quality of the studies are listed in Table 4, and are explained in the topic: Assessment of the methodological quality of the studies.

**Table 4:** Methodological evaluation of studies using the PEDro scale

Author/year	1	2	3	4	5	6	7	8	9	10	11	revisor 1 Total
Li et al., (2022) [14]	X	1	1	1	0	0	1	1	1	0	1	7/10
PEHLIVAN et al., (2022) [15]	X	1	0	1	0	0	0	1	1	1	1	6/10
Nambi et al., (2022) [16]	X	1	1	1	0	0	1	1	1	1	1	8/10
Foged et al., (2021) [17]	X	1	1	1	1	0	0	1	1	0	1	7/10
Giardini et al., (2022)	X	0	0	1	0	0	0	1	1	1	1	5/10

1–11 PEDro scale evaluation criteria

## Discussion

In Li et al. (2022) [14], they performed a 6-week telerehabilitation program that was compared with control (i.e., short educational instruction) for COVID-19 survivors after hospital discharge. The rehabilitation protocol consisted of home exercises with sessions that lasted from 40 to 60 min through a smartphone application. Participants were instructed to monitor and record oxygen saturation before and after exercise using a finger pulse oximeter. Telemetry was used with the aim of monitoring the heart rate of the study volunteers during the execution of the proposed exercises. The application used in the study provided participants with instructions on telemetry adjustments, execution of the exercise program, and notified participants regarding exercise start times. Sessions were conducted via smartphone or voice calls on a smartphone communication application. The application also allowed participants to send their feedback regarding the usability of the application. This study demonstrated superiority of the telerehabilitation program over no rehabilitation for 6MWD, LMS and physical HRQoL.

Pehlivan et al. (2022) [15] carried out a study that consisted of a 6-week telerehabilitation program with breathing exercises, lower and upper limb exercises, walking exercises and wall squats. This protocol was compared with no intervention in post-COVID-19 patients after hospital discharge. Participants in the experimental group were instructed to perform exercise sessions three times a week through videoconferencing. Participants in the control group received educational material with information about the disease and some basic exercises that could be performed at home. All participants were evaluated by video calls without face-to-face meetings. The authors reported that, in general, the online exercise program has a positive effect on the physical performance of Covid 19 cases, corroborating the study by Li et al. who showed that a telerehabilitation program over no rehabilitation is superior in post-COVID-19 patients. 19. The combination of these two studies suggests that exercise performed via telerehabilitation may benefit individuals with post-COVID-19 sequelae.

In the study by Nambi et al. (2022) [16], a study was conducted with elderly men with symptoms of sarcopenia post-CONVID-19 to compare the clinical and psychological effects of low- and high-intensity aerobic training combined with resistance training for 8 weeks. The authors observed that there was a significant difference between the groups. At the end of six months of follow-up, the low-intensity aerobic training group was superior to the high-intensity aerobic training group, but there was no difference in the muscle mass variable in both groups. In view of the results found, the authors reported that low-intensity aerobic training exercises are more effective in improving clinical (muscular strength) and psychological measures (kinesiophobia and quality of life) than high-intensity aerobic training in post-COVID sarcopenia 19. This study was the only one that compared sarcopenia in isolation in post-COVID-19 patients.

Foged et al. (2021) [17], performed a randomized crossover study, where they compared three high-intensity interval training (HIIT) protocols, the duration of the three HIIT protocols was combined with the duration of the 38-min exercises. Patients participated in the study reporting the feeling of weakness when performing exercises after COVID-19, the results demonstrate that the use of the three protocols is considered safe and tolerable, with no adverse events occurring during training. The study highlights that the use of HIIT protocols should be considered and used in future clinical trials in the rehabilitation of post-COVID-19 patients. Considering the small sample size and that this study was the only selected one that tested post-COVID-19 HIIT protocols in a supervised way, the authors also investigated the fidelity, tolerability and safety of three HIIT protocols with different working periods. or intensity, allowing comparisons between protocols. However, it was not possible to compare the results of this study with others in this review due to differences in the variables analyzed.

The study by Giardini et al. (2022) aims to understand the deficiencies and outline a rehabilitation protocol aimed at the specific needs of patients, the authors carried out an unprecedented study and compared the static and dynamic balance of healthy people, of individuals with lung disease and individuals with COVID-19. Static balance with eyes open and closed was assessed using a stabilometric platform and dynamic balance using the Mini BestTest and the Timed Up and Go. The findings show that both groups formed by patients showed worse performance in both static and dynamic balance, highlighting the importance not only of respiratory rehabilitation, but also of protocols aimed at balance and mobility training, preventing patients from suffering episodes of fatigue in the future. falls related to balance deficit.

In general, the studies described in this review evaluated physical exercises in post-COVID-19 patients, with the objective of evaluating the following variables: physical performance, sarcopenia, sensation of weakness, mobility and balance, in addition to the tolerability and benefits of rehabilitation protocols for individuals who have had COVID-19 infection. Of the selected studies, two were carried out through platforms for telerehabilitation and three in face-to-face consultations. In conclusion, exercise programs performed can improve functional capacity and quality of life compared to the absence of rehabilitation or compared to control groups, in people with post-COVID-19 conditions.

Through the evaluation of the selected studies, the importance of rehabilitation and improvement of the quality of life of patients who underwent physical therapy treatment due to changes in COVID-19 was observed. With this, we can certify the resulting impact of the rehabilitation process on the clinical treatment of the patient, improving and helping even more in the treatment of other areas of health such as medicine and multidisciplinary areas, in addition to stimulating the search for knowledge and research in this topic.

The most important limitation of this study is the high heterogeneity of the selected studies, especially among the different evaluation methods used. Assessing methodological quality in the reviewed studies was difficult because different designs were identified, due to this limitation the PeDro scale was added. Another important limitation is that not all studies have reported in detail the functional and mainly motor sequelae, nor the detailed treatment protocols of the research participants, making it impossible to know the real situation of the functional capacity of individuals who had COVID-19. Therefore, our study suggests conducting new clinical trials that seek to elucidate this limitation so that in the future it will be possible to outline individualized rehabilitation protocols focused on the changes presented by this population.

## Conclusion

In conclusion, physiotherapy is an important and necessary intervention for the rehabilitation of sequelae from COVID-19. After evaluation, it was observed that the protocols used in studies for the rehabilitation of individuals with conditions inherent to COVID-19 that were explained in this review, present safe results. However, it is important to carry out new studies that present greater methodological rigor, larger sample numbers and analysis of other important outcomes such as: patient satisfaction with rehabilitation, level of functional independence and guidelines for post-COVID-19 motor exercise protocols, since in the literature there is already an important number of studies that report respiratory and hospital rehabilitation. These studies can help verify the safety and efficacy of physical therapy rehabilitation in individuals with functional sequelae from COVID-19, making this rehabilitation process more effective and with reliable results.

### Supplementary Information

**Additional file 1.**

**Additional file 2.**

## Data Availability

The entire dataset supporting the results of this study was published in the article itself.
