# Supplementary material for: Physical therapy rehabilitation after hospital discharge in patients affected by COVID-19: a systematic review
Source: BMC Infect Dis. 2023 Aug 16;23:535. doi: 10.1186/s12879-023-08313-w (PMC10429071; doi:10.1186/s12879-023-08313-w)
Supplement: Supplementary file 1 — Additional file 1. [file 12879_2023_8313_MOESM1_ESM.pdf]

CONSULTED DATABASES

Bases	Number	excluded by duplicity	excluded by title	excluded by summary	full text excluded	accepted
PubMed	174	2	128	25	17	2
Scielo	6	1	1	1	3	0
Science Direct	132	5	72	32	22	1
BVS	31	3	14	9	4	1
PEDro	21	3	14	2	1	1
Total	364	14	229	69	47	5
